# The Secretome of Brain Endothelial Cells Exposed to the Pyrrolizidine Alkaloid Monocrotaline Induces Astrocyte Reactivity and Is Neurotoxic

**DOI:** 10.3390/toxins17020065

**Published:** 2025-02-01

**Authors:** Letícia Oliveira Santos, Julita Maria Pereira Borges, Juliana Lago Leite, Mauricio Moraes Victor, Adriana Lopes da Silva, Cleonice Creusa dos Santos, Victor Diógenes Amaral da Silva, Ravena Pereira do Nascimento, Silvia Lima Costa

**Affiliations:** 1 Laboratory of Neurochemistry and Cellular Biology, Health Sciences Institute, Federal University of Bahia, Av. Reitor Miguel Calmon s/n Vale do Canela, Salvador 40231-300, BA, Brazil; leticiaoliveira.vet@gmail.com (L.O.S.); jmpborges@uesb.edu.br (J.M.P.B.); cleonicemev@gmail.com (C.C.d.S.); vdsilva@ufba.br (V.D.A.d.S.); 2 Department Health of Science, State University of Southwest of Bahia (UESB), Estrada do Bem Querer Km 04, Vitória da Conquista 45083-900, BA, Brazil; 3 Department of Organic Chemistry Chemistry Institute, Federal University of Bahia, R. Barão de Jeremoabo, 147—Ondina, Salvador 40170-115, BA, Brazil; july.lago@gmail.com (J.L.L.); mmvictor@ufba.br (M.M.V.)

**Keywords:** monocrotaline, neurotoxicity, blood–brain barrier, brain endothelial cells, astrocytes

## Abstract

Monocrotaline (MCT) has well-characterized hepatotoxic and pneumotoxic effects attributed to its active pyrrole metabolites. Studies have previously shown that astrocytes and neurons are targets of MCT, and that toxicity is attributed to astrocyte P450 metabolism to reactive metabolites. However, little is known about MCT toxicity and metabolism by brain endothelial cells (BECs), cells that, together with astrocytes, are specialized in xenobiotic metabolism and neuroprotection. Therefore, in the present study, we evaluated the toxicity of MCT in BECs, and the effects on astrocyte reactivity and neuronal viability in vitro. MCT was purified from *Crotalaria retusa* seeds. BECs, obtained from the brain of adult Wistar rats, were treated with MCT (1–500 µM), and cell viability and morphology were analyzed after 24–72 h of treatment. Astrocyte/neuron co-cultures were prepared from the cortex of neonatal and embryonic Wistar rats, and the cultures were exposed to conditioned medium (secretome) derived from BECs previously treated with MCT (100–500 µM, SBECM100/500). MCT was not toxic to BECs at the concentrations used and induced a concentration-dependent increase in cell dehydrogenase after 72 h of treatment, suggesting resistance to damage and drug metabolism. However, exposure of astrocyte/neuron co-cultures to the SBECM for 24 h induced changes in the cell morphology, vacuolization, and overexpression of GFAP in astrocytes, characterizing astrogliosis, and neurotoxicity with a reduction in the length of neurites labeled for β-III-tubulin, effects that were MCT concentration-dependent. These results support the hypothesis that MCT neurotoxicity may be due to products of its metabolism by components of the BBB such as BECs and astrocytes, which may be responsible for the brain lesions and symptoms observed after intoxication.

## 1. Introduction

Plants are true sources of chemical products, called metabolites, whose main function is always to produce them for their own benefit. However, when these substances come into contact with the animal or human body, they can cause intoxication. Monocrotaline (MCT) is a pyrrolizidine alkaloid (PA) and is considered to be the most important plant toxin affecting animals and humans [1]. Studies have characterized the presence of PAs in plants and in human and animal foods such as milk, honey and pollen, herbal tea alfalfa-based horse feed, and in rabbit feed, thus raising the question of the possibility of a downstream contamination [2,3,4,5,6,7,8,9]. In addition, cereal grains and their preparations can be contaminated with seeds of PA-rich plants and accidentally ingested by animals and humans in food and in phytomedicine [10,11]. Monocrotaline and monocrotaline-type PAs, such as tricho-desmine, fulvene, and retusamine, are present in plants of the genus *Crotalaria* spp. (Fabaceae family) [4], typical of tropical and subtropical regions [1]. More recently, the occurrence of PAs in grain cultivations has been reviewed by Schramm et al. (2019) [12]. *Crotalaria retusa* is a toxic plant, being popularly known as “rattlesnake rattle or snake rattle”, characterized by high drought resistance, invasiveness, easy presence in pastures and cereal plantations, and high content of MCT, its main toxin, in its seeds and aerial parts [13].

Intoxication and metabolism of MCT have been studied for a long time and, as revised by us [14], MCT is converted by the hepatic cytochrome P450 (CYP) system to the very unstable metabolites *N*-oxides and dehydromonocrotaline (DHMCT). These in turn can be hydrolyzed to 6,7-dihydro-7-hydroxyl-1-hydroxymethyl-5H-pyrrolizine (DHP), one of the main active metabolites, or can be converted to dehydro alkaloids, which can conjugate with glutathione (GSH), the endogenous peptide involved in the neutralization of xenobiotics, to form the enantiomers 7-glutationyl-6,7-dihydro-1-hydroxymethyl-5H-pyrrolizine (7-GS-DHP) and 7,9-diGSH- DP, which are capable of alkylating nucleophilic macromolecules such as DNA and proteins. In a study by Honorio et al. (2012) [15], MCT administered in drinking water induced hepatotoxicity, pneumotoxicity, and nephrotoxicity in mice. However, as first described by Schoental and Head (1995) [16], intraperitoneal administration of MCT (5, 50, or 100 mg/kg) in mice induced cellular lesions in the parahippocampal cortex and in the hippocampus regions. Metabolites of MCT and other Pas, such as trichodesmin, dehydrotricodesmin, and dehydromonocrotalin, have already been found and measured in the brain of experimentally intoxicated rats [17], suggesting that these molecules can cross the blood–brain barrier (BBB) and that the neurological signs observed in intoxicated animals may be a result of the efficient metabolism of APs into active components in CNS cells. 

Astrocytes, together with brain endothelial cells (BECs), form the blood–brain barrier (BBB), which regulates the entry of substances into the brain, and confers selectivity [18]. Our previous in vitro studies showed that MCT and its metabolite DHMCT induce astrocytic reactivity [19], including a broad genotoxic effect [20]. In addition, MCT showed toxic effects in a neuron–glia system, with alterations in neuronal and astrocytic cytoskeletal proteins [21]. The involvement of the astrocyte CYP1A1 isoform in the cytotoxicity of MCT associated with GSH depletion has also been demonstrated [22]. As the main constituent of the BBB, BECs are also the main drug-metabolizing cells in the CNS that, together with an effective antioxidant defense system, form a metabolic barrier that protects the brain against xenobiotic entry and reduces the accumulation of neurotoxic proteins, as recently reviewed by Wei et al. (2023) [23]. It therefore plays an important role in the detoxification of pharmacological agents, either by producing mostly inactive products or by generating reactive metabolites that are in turn released from cells and excreted from the body [24,25]. These reactive metabolites can lead to organic disorders simply by altering vascular permeability, resulting in a loss of homeostasis and consequently pathogenesis in the CNS.

The imbalance of the BBB predisposes the CNS to excitotoxicity and neurodegenerative diseases; therefore, it is important to investigate the involvement of these cells in the metabolism and neurotoxicity of MCT. Recently, we observed that MCT (109 mg/Kg), via acute oral administration in rats, induced an anxiolytic-like effect, and hyperemic vascular structures in the hippocampus, parahippocampal cortex, and neocortex, mild perivascular edema in the neocortex, hemorrhagic focal area in the brainstem, and edema in the thalamus [26]. Additionally, MCT intoxication induced astrocyte reactivity and up-regulation of mRNA expression of neuroinflammatory mediators, particularly interleukin-1 beta (IL1-β) and the chemokine (C-C motif) ligand 2 (CCL2) in the hippocampus and cortex, and down-regulation of mRNA expression of neurotrophins of HGDF and BDNF in the cortex, highlighting CNS damage after acute intoxication [26]. However, the involvement of BECs in MCT metabolism and neurotoxicity is not yet understood. Hence, in this study, we evaluate the toxicity of MCT in an in vitro model of primary cultures of rat BECs and its relationship with neurotoxicity and astrocytic reactivity in cortical glia–neuron co-cultures.

## 2. Results

**Figure 1:**
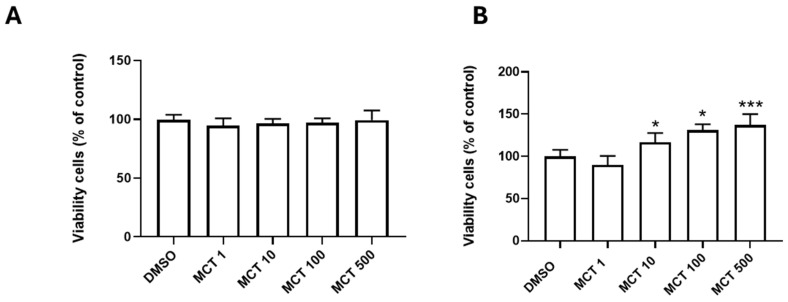
Effect of MCT on the viability of BECs by MTT test. Cells were maintained under control conditions (0.5% DMSO) or treated with monocrotaline (MCT) at concentrations from 1 to 500 µM. (**A**,**B**) Analysis of dehydrogenase activity after 24 h and 72 h treatments; data were analyzed by one-way ANOVA and by the nonparametric Mann–Whitney test; (*) refers to *p* < 0.05 when comparing treatments and DMSO control; (***) refers to *p* < 0.001 when comparing treatments and DMSO.

Firstly, we evaluated the effect of MCT (1–500 µM) on BEC viability by the MTT test, 24 and 72 h post-treatments. At these times of exposure, the values for the vehicle control samples (0.5% DMSO) were set at 100% for all treatments; statistical significance was * *p* < 0.05 compared to the control (Figure 1). After 24 h of treatment, MCT, at the adopted concentrations, was not toxic to BECs (Figure 1A). However, after 72 h of treatment with MCT (Figure 1B), there was a significant and concentration-dependent increase in the cellular dehydrogenase activity, suggesting resistance to damage and drug metabolism.

**Figure 2:**
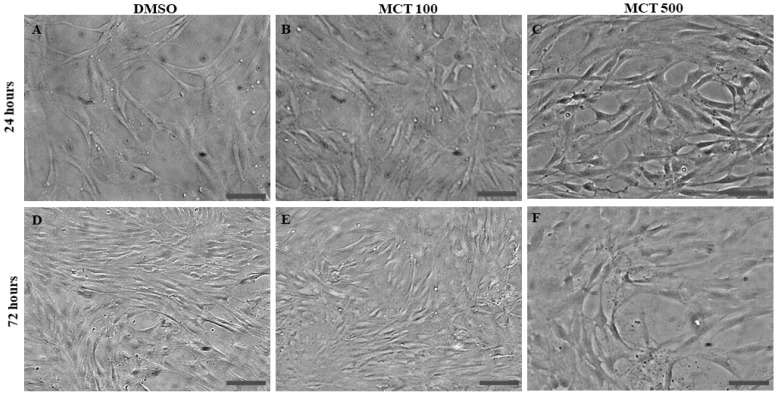
Analysis of brain endothelial cell (BEC) morphology after 24 h and 72 h culture in control conditions (0.5% DMSO) or treated with alkaloid monocrotaline (MCT) by phase-contrast micrograph. (**A**–**C**) Photomicrographs after 24 h treatments were taken using objective 20×, scale bar = 100 µm; (**C**,**D**) photomicrographs after 72 h treatments were taken using objective 10×, scale bar = 100 µm. (**A**,**D**) Control conditions (0.05% DMSO); (**B**,**E**) BECs treated with MCT at 100 µM (MCT100); (**C**,**F**) BECs treated with MCT at 500 µM (MCT500), respectively. Changes in cell morphology and increase in cell density are evident in treated cultures compared to controls.

**Figure 3:**
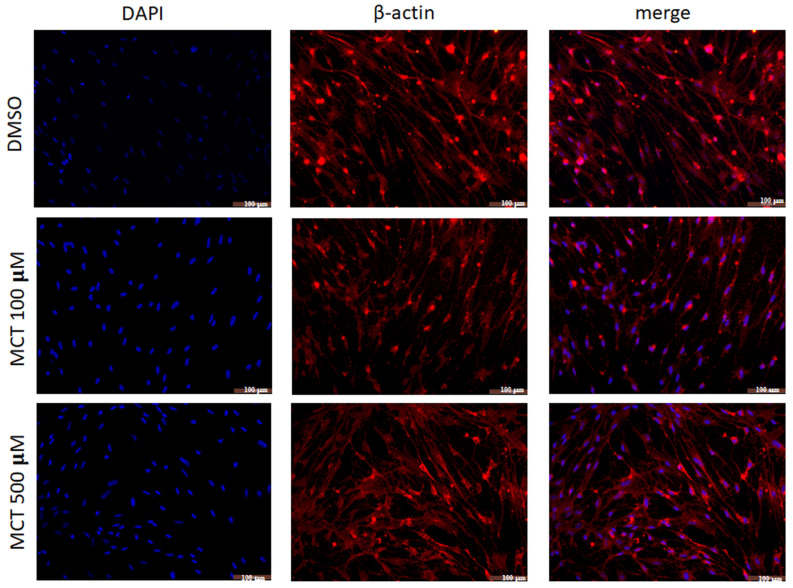
Analysis of brain endothelial cells (BECs) morphology after 24 h in control conditions (0.5% DMSO) or treated with alkaloid monocrotaline (MCT) by immunocytochemistry for the β-actin protein (red). Photomicrograph cultures in control conditions (0.5% DMSO), or after 24 h treatment with MCT at 100 µM or 500 µM; nuclear chromatin was stained by DAPI (blue); objective 20×, scale bar = 100 µm. The disruption of actin filaments and the presence of more cell nuclei are evident in treated cultures compared to the control.

To investigate the morphology and cell density in BEC cultures, they were analyzed by phase microscopy and by immunocytochemical labeling for the protein α-actin, component of the cytoskeleton (Figure 2 and Figure 3, respectively), under different conditions and after 24 and 72 h after treatments. It was observed that the BECs under the control conditions form a typical monolayer of cells with a predominantly bipolar shape with few processes, a phenotype maintained 72 h after culture, although an increase in cell density was observed in the cultures (Figure 2A,C). BECs treated with MCT at the lowest tested concentrations of 1 and 10 µM also preserved the typical morphology 24 h after treatments. However, many cells acquired a distinct phenotype, presenting a multipolar phenotype and contracted cell body when treated with MCT at 100 µM and 500 µM (Figure 2B,C). On the other hand, 72 h after exposure to 100 and 500 µM MCT (Figure 2 E,F), an increase in cell density was observed in the monolayer compared to the control; most cells presented a bipolar and elongated phenotype but also many cells presented a multipolar phenotype and a contracted cell body, findings that also suggest changes in metabolism and proliferation.

Changes in the morphology of BECs after treatment with MCT were confirmed by immunocytochemical labeling for the α-actin. It was possible to observe the typical bipolar phenotype under control conditions (Figure 3) and an increase in the proportion of cells with a multipolar phenotype and contracted cell body after 24 h after treatment with MCT at concentrations of 100 and 500 µM (Figure 3). Also, there was observed discontinuity in actin immunostaining, indicating possible protein disruption induced by treatment with the alkaloid.

**Figure 4:**
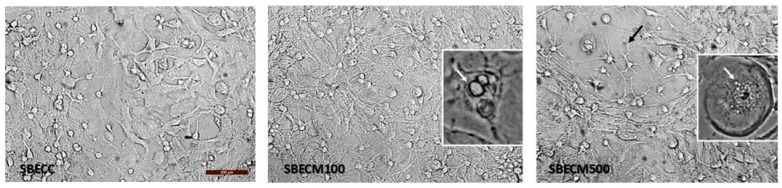
Analysis of cell morphology after 24 h exposure of neuron/astrocyte co-cultures to the conditioned medium from BECs in control conditions (SBECC) or treated with MCT at 100 µM (SBECM100) and 500 µM (SBECM500) in the morphology of cells. Analysis by interference microscopy; objective 20×; scale bar = 200 µm. Large, stellated cells with several cellular processes and vacuoles (white arrows, in highlights), as well as various cells with a very contracted cell body and fine processes (black arrow), are visible after treatment with the secretome of BECs exposed to MCT. A decrease in the cell density of the monolayer compared to control cultures exposed to the untreated BECs (SBECC).

To analyze the effect of the response of BECs to the treatment with the alkaloid MCT on the phenotype and viability of astrocytes and neurons, co-cultures of neurons and astrocytes were treated with the conditioned medium (secretome) generated by BECs in control conditions (SBECC) or with the conditioned medium generated by BECs 24 h after treatment with MCT 100 µM or 500 µM (SBECM500/100). The analysis of cell morphology by interference microscopy (Figure 4) revealed that cultures treated with SBECC maintain typical parameters of a neuron network on the glial cell monolayer, constituted mostly of astrocytes (>90%), as described previously. However, in cultures treated with SBECM100, an increase in the proportion of cells on the surface of the cultures was observed, possibly phagocytic cells. These events were also observed in co-cultures exposed to SBECM500, but areas without cells were also evidenced, possibly resulting from cell body contraction and elongation of astrocyte processes, in addition to cells with a small cell body and various processes in these areas. Moreover, the co-cultures treated with SBECM100 had increased the cytoplasmic density, as well as an increased number of cellular processes and vacuoles; the co-cultures treated with SBECM500 showed a reduction in cell density and increased vacuolation, indicating that the effects were amplified due to the increase in the concentration of MCT adopted in the treatment of BECs.

**Figure 5:**
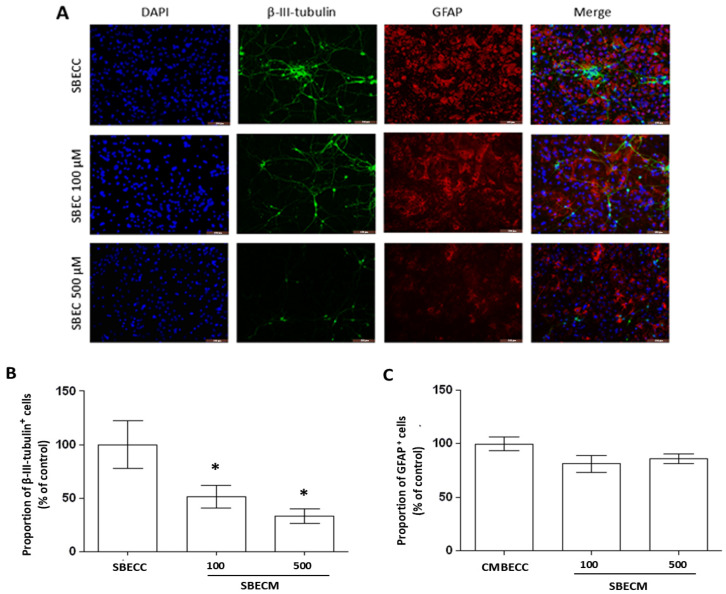
Analysis of astrocyte and neuron morphology after 24 h exposure to the conditioned medium from BECs exposed to MCT. (**A**) Photomicrography of immunocytochemistry for GFAP (red) and β-III-tubulin (green) markers in neuron/astrocyte co-cultures treated for 24 h with conditioned medium from BECs in control conditions (SBECC) or treated with monocrotaline at 100 (SBECM100) or at 500 µM (SBECM500); nuclei were stained with 4′,6-diamidino-2-phenylindole (DAPI, blue); obj. 20×; scale bar = 100 µm; the activation of astrocytes was evidenced by the increase and changes in the pattern of GFAP expression; a strong reduction in β-III-tubulin staining in cell bodies and along neuron neuritis after treatment with conditioned medium derived from BECs treated with MCT was observed; (**B**,**C**) proportion of β-III-tubulin- and GFAP-positive cells in co-cultures in control and treated with conditioned medium from BECs treated with MCT; the results were analyzed for variance and Tukey post-test, and results expressed as means of the percentage of GFAP- or β-III-tubulin-positive cells/total cells ± standard deviation; * *p* < 0.05 compared to control in three independent experiments.

To assess the astrocyte and neuron morphology in co-cultures exposed to SBEC, immunocytochemistry was performed for the glial fibrillary acidic protein (GFAP), specific to the cytoskeleton of astrocytes and a marker of astrogliosis, and for the protein β-III-tubulin, a specific component of the cytoskeleton of neurons (Figure 5). In the control conditions of co-cultures exposed for 24 h to SBECC, astrocytes with a typical flat and polygonal phenotype were observed, with some cells showing short cellular processes and the network of neurons and their neurites evidenced with the β-III-tubulin protein expressed in the cell body and in neurites with a filamentous appearance. However, in co-cultures exposed to SBECM100 or SBECM500, changes in the morphology of astrocytes were evidenced with an increase in the expression of GFAP, also presenting more compact cell bodies. However, exposure to SBECM100 also induced a reduction in the size and in the number of neurites as well as failures in the polymerization of these proteins along the structure of neurons, an effect amplified in cultures exposed to SBECM500. Quantification of β-III-tubulin-positive cells also evidenced a significant and concentration-dependent reduction in the proportion of neurons in the co-cultures exposed to SBECM100 or SBECM500 without significantly affecting the proportion of GFAP-positive astrocytes (Figure 5B and C, respectively).

## 3. Discussion

Astrocytes and BECs from the BBB have a highly active drug metabolism and detoxification system necessary to protect the brain against potentially toxic metabolites or xenobiotics [27,28].

In the present work, the effect of MCT in BEC cultures was evaluated, and it was observed that it was not toxic at the concentrations adopted but induced an increase in the density of the cells’ monolayer after 24 h after treatments with the alkaloid at the higher concentrations (100 and 500 µM), suggesting resistance to damage and alkaloid metabolism and proliferation. Considering that the metabolites of PA have long demonstrated being capable of alkylating macromolecules as cytoskeletal proteins as actin in pulmonary artery endothelial cells [29], the present study investigated the possible alterations in the expression of actin protein in BECs treated with MCT. In previous studies, alterations in the expression of cytoskeletal proteins were also evidenced in astrocytic cells directly treated with MCT or its metabolite DHMCT, affecting the pattern of glial fibrillary acidic protein (GFAP) and microtubule-associated protein (MAP) expression [20,21]. Moreover, changes in the pattern of GFAP and β-III-tubulin expression and steady-state levels after MCT treatment, with a dose-dependent and time-dependent intense down-regulation and depolarization of neuronal β-III-tubulin, were also observed in astrocyte/neuron co-cultures [19]. Here, we observed the presence of thinner and more sparse cellular actin processes and discontinuity in immunostaining, indicating possible protein disruption induced by treatment with MCT, reinforcing the property of the alkaloid to alkylate structural proteins that can result in a loss of or reduction in functionality.

In order to analyze the effect of the products of the response of BECs to the exposure to MCT on astrocyte response and neuron viability, co-cultures were treated with the secretome from BECs treated with the alkaloid. Interference microscopy showed the presence of morphological changes and cytoplasmic vacuoles in astrocyte/neuron co-cultures treated with conditioned medium from BECs exposed to 100 µM and 500 µM MCT. Previously, we demonstrated that the treatment of astrocytes with MCT or its metabolite DHMCT induces DNA loss by chromatin condensation and fragmentation, and cytoskeletal stability in astrocytes treated with the highest concentrations of 500 µM [19,30]. However, in these works by Barreto et al. (2006) [30] and by Barreto et al. (2008) [19], cytoplasmic vacuoles in the primary cultures of astrocytes treated directly with MCT with the main metabolite DHMCT were not observed. Interestingly, Pitanga et al. (2011) [21] showed vacuolization in astrocyte/neuron co-cultures treated with 100 µM MCT. This assumes that the MCT metabolism and toxic properties are dependent on interactions between neurons and astrocytes.

Astrocytes provide nutritional and structural support for neurons and direct the growth of neurites, serving as a guide [31]. β-III–tubulin is the main structural protein of neurons; therefore, it is a marker of specific microtubules of neurons [32]. In the CNS, astrocyte reacts to insults of different origins with functional and morphological alterations, a response named reactive astrogliosis, in view of protecting and reestablishing homeostasis [33,34], and the GFAP, which constitutes the intermediate filaments of astrocytes, is a consistent marker of astrogliosis also associated with BBB dysfunction [33,34,35,36]. In the present study, when analyzing astrocytes and neurons through the immunocytochemical technique for GFAP and β-III-tubulin, in co-cultures exposed to the SBEC treated with MCT, an increase in the expression of GFAP with a reduction in neurites after 24 h of exposure was observed. Moreover, interruptions in the expression of β-III-tubulin were observed in these conditions, suggesting a failure in the polymerization of this important structural protein in neurons. Changes in the morphology of GFAP expression and intense cytoplasmic vacuolization in astrocytes was also observed after exposure for 24 h with conditioned medium from BECs treated with MCT, corroborating the findings of Pitanga et al. (2011) [21], which also demonstrated a strong decrease in the expression of β III-tubulin and protein disruption in astrocyte/neuron co-cultures treated directly with MCT. The changes observed in astrocytes and neurons in the present study were possibly induced by products of MCT metabolism by BECs, which may induce astrogliosis and interfere with the dynamics of the stabilization of microtubular proteins impacting on the viability of neurons.

Accidental and experimental MCT- or *Crotalaria* spp.-intoxicated animals have presented neurological clinical signs mainly interpreted as the consequence of hepatic encephalopathy [37,38]. However, MCT and metabolites were detected in the brain of intoxicated animals [17], which suggests that these molecules can cross the BBB. In our previous study [26], a remarkable overexpression of GFAP in astrocytes around vessel-like structures in the cerebral cortex of MCT-intoxicated rats, associated with cerebrovascular alterations, such as hyperemia, perivascular edema, and hemorrhage in different brain areas, evidencing that astrogliosis is part of MCT in in vivo intoxication.

## 4. Conclusions

Together with our previous finding that demonstrated that astrocytes and neurons from the brain are sensitive to MCT and its metabolites. The results presented by this work support the hypothesis that MCT neurotoxicity may be due to products of its metabolism by components of the BBB, such as BECs and astrocytes, responsible for the brain lesions and symptoms observed after intoxication. Further studies, including experimental intoxication with a focus on BECs, will help to elucidate MCT and the consequences of its metabolism in the brain.

## 5. Materials and Methods

### 5.1. Monocrotaline (MCT) Purification

MCT was isolated from lyophilized aqueous extract of *C. retusa* seeds according to the method of Culvenor and Smith (1957) [13] with modifications, except that hexane was used instead of petroleum ether and possible *N*-oxide forms were not converted to free bases, as previously described [26,30]. Firstly, lyophilized aqueous extract was passed through a Soxhlet extractor in solvents of increasing polarity. The freeze-dried aqueous extract was passed in hexane for 16 h, to remove lipophilic oils and fats, and then the substrate was washed in ethanol for 16 h, to solubilize any alkaloids present. The resulting ethanolic extract was filtered and acidified with 5% H_2_SO_4_, to convert the alkaloids into salts. The solution was alkalinized to pH 10.5, with NH_4_OH, which returned the alkaloids to their free form. The alkaloids were then extracted with chloroform and concentrated under vacuum until crystallization. Crystals were then dissolved in heated methanol and, after cooling the solvent, the pure MCT was recrystallized and deposited on the bottom of the bottle. The characterization of MCT was performed by 13C and 1H nuclear magnetic resonance, in addition to the infrared spectrum. The yield was 2.8 g MCT per 100 g lyophilized aqueous extract. For the experiments, MCT was dissolved primarily in dimethylsulfoxide (DMSO), forming a stock solution at 100 mM, and during treatments, it was dissolved directly in the culture medium at concentrations ranging from 1 to 500 µM.

### 5.2. Cultures of BEC

Pure primary cultures of brain endothelial cells were obtained from young adult male Wistar rats weighing 200–300 g according to (Bayol-Denizot et al., 2000) [39] with modifications by Borges (Borges, PhD diss., 2018). Rats were killed by decapitation and the brains were rapidly removed after craniotomy in a sterile field and cooled to 0–2 °C in serum-free supplemented DMEM/HAM F12 homogenization medium. The cortices were gently homogenized (in a Dounce homogenizer) in DMEM/HAM F12-supplemented medium, without fetal bovine serum (FBS), centrifuged at 600× *g* for 5 min, at 4 °C; the precipitated mass was resuspended in a 1 mg/mL collagenase (EC 3.4.24.7)/dispase (EC 3.4.24.4) solution diluted in the same medium as described above for 1 hour at 37 °C for enzymatic dissociation. Then, the homogenate was centrifuged again at 1000× *g*, at 4 °C, for 10 min. The resulting pellet was resuspended in 26% (*v*/*v*) FBS solution in Hanks’ balanced salt solution (HBSS), shaken vigorously for 1 min, and then centrifuged at 2500× *g* for 10 min, at 4 °C. To the obtained sediment, 1 mg/mL of collagenase/dispase solution was added and incubated for 3 h, at 37 °C, with gentle shaking. After the incubation period, the homogenate was centrifuged at 1000× *g* for 5 min, at 4 °C. To separate the CECs, the resulting sediment was dissolved in 2 mL of HBSS 1× and added to the Percoll gradient, then centrifuged at 2000× g for 15 min. The BECs were then homogenized in supplemented DMEM/HAM F12 medium and distributed for cultivation in 75 cm^2^ (TPP) flasks covered with 20 µg/cm^2^ rat tail collagen for cell expansion at 37 °C in an incubator with CO_2_ at 5%. The first change in medium was made after 24 h and subsequent changes were made every 48 h, until the cells reached confluence. For experiments, the cells were trypsinized and plated on 96-well, 24-well, or 06-well plates, at a density of 3.1 × 10^4^ cells/cm^2^, according to the different analytical procedures to be performed.

### 5.3. Primary Co-Culture of Astrocytes/Neurons

Primary cultures of glial cells enriched for astrocytes have been described previously [21]. They were obtained from the cerebral hemispheres of one-day postnatal Wistar rats, which were aseptically isolated and the meninges removed. The cortex was dissected and then gently pressed through a sterile 75 mm. Cells were suspended in DMEM/HAM F12 medium (Gibco, Grand Island, NY, USA), supplemented with 100 IU/mL penicillin G, 100 mg/mL streptomycin, 2 mM L-glutamine, 0.011 g/mL pyruvate, 10 µM FCS %, 3.6 g/L HEPES, and 12 mM glucose (Gibco, Grand Island, NY, USA) and cultured in 24-well plates at a cell density of 2 × 10^5^/well, and maintained in a humidified atmosphere with 5% CO_2_ at a temperature of 37 °C. The culture medium was changed every 2 days, and the cells were cultured for 15 days. At this time, neurons were obtained in the cerebral hemispheres of 15-to-18-day old Wistar rat embryos using the same method described above and seeded over the astrocyte culture monolayer at a density of 1 × 10^5^/well. The cultures were maintained in supplemented DMEM/HAM F12 in a humidified atmosphere with 5% CO_2_ at 37 °C for 8 days. After this time, the cells were treated with brain endothelial cell-conditioned medium for 24 h each, as described below.

### 5.4. Evaluation of MCT Cytotoxicity in BECs

Cell viability was analyzed through the activity of mitochondrial dehydrogenases by the tetrazolium salts reduction test, which is performed by a sensitive, quantitative, and reliable colorimetric measurement, used to measure cell viability, proliferation, and metabolic activity. Its principle is based on the ability of living cell mitochondrial dehydrogenases to convert 3-(-dimethylthiazolyl-2)-2,5-diphenyltetrazolium bromide (MTT), a yellow substrate, from formazan crystals to a violet-colored substrate (Hansen et al., 1989). For this, BECs were cultivated in polystyrene plates (TPP) containing 96 wells (3.1 × 10^4^ cells/cm^2^) over a collagen layer and after 24 and 72 h submitted to treatment with MCT at concentrations of 1, 10, 100, and 500 µM. After the treatment period, an MTT solution (Sigma, St Louis, MO, USA, EUA) at a final concentration of 1 mg/mL diluted in DMEM/HAM F12 was added to the culture wells (100 μL/well) and incubated for 2 h. After this time, 100 μL of lysis solution (SDS—20% sodium dodecyl sulfate and 50% dimethylformamide, pH 4.7) was added for 24 h at 37 °C to dissolve the formazan crystals. Then, absorbance was measured at a wavelength of 595 nm in a spectrophotometer in a microplate reader (Thermo Plate, model TP Reader—type B, Varioskan, Term Fisher Scientific, Waltham, MA, USA, EUA). The results were presented as the percentage of viability (mean and standard deviation) in relation to the negative control with DMSO (0.5%), considered as 100%, after its comparative analysis, with the negative control containing only culture medium.

### 5.5. Obtaining the Conditioned Media

Conditioned media (secretoma) of BECs were obtained from cultures of BECs under control conditions (SBECC), treated with MCT at 100 µM (SBECM100), and treated with MCT at 500 µM (SBECM500) or 24 h. After this time, the culture medium was then recovered, centrifuged at 1000× *g* for 10 min, to eliminate any cellular debris, and immediately used or stored in aliquots at −20 °C for later use. Astrocyte/neuron co-cultures were treated for 24 h with CMBEC at the different conditions.

### 5.6. Morphology and Immunocytochemical Analysis

Morphological changes were studied by phase-contrast microscopy and immunocytochemical patterns for cytoskeletal proteins of astrocytes (glial fibrillary acidic protein, GFAP) and neurons (β-III tubulin), in addition to the β-actin protein, which was adopted for the analysis of BEC morphology. These proteins are highly conserved and are involved in cell motility, structure, and integrity. Cells were washed three times with PBS, fixed and permeabilized with ice-cold methanol at −20 °C for 10 min. Nonspecific antibody binding was blocked by pre-incubating the cells with 3% bovine serum albumin (BSA) in PBS.

Immunocytochemical labeling of BEC was performed by incubating with polyclonal antibodies produced in rabbits to β-actin protein (1:200 Sigma) in PBS for 3 h and, after three washes with PBS, the cells were incubated with secondary antibodies specific for IgG from rabbits conjugated with fluorochrome Alexa Fluor 594 (1:200 Term Fisher Scientific, Waltham, MA, USA, EUA), for 1 hour. Then, after three washes with PBS, the cells were incubated for 30 min with 4,6-diamino 2-phenylindole (DAPI, 5 µg/mL), an intercalating agent of deoxyribonucleic acids, to identify the nuclear chromatin. Finally, after three washes with PBS, coverslips were mounted with the addition of *N*-propyl gallate to prevent loss of fluorescence.

For the identification of astrocytes and neurons, co-cultures, seeded in 24-well culture plates, also on cover slips, were washed three times with PBS and fixed with cold methanol at 20 °C for 10 min after treatments with MC. Cells were initially incubated with GFAP-specific rabbit polyclonal antibodies (1:500 in PBS, Boehringer, Manheim, Germany) or with mouse monoclonal antibodies specific for β-III tubulin (1:500 in PBS, Biolegend, San Diego, CA, USA, EUA) for 3 h in a darkroom. After the incubation time, the cells were washed three times with PBS and then incubated with secondary antibodies specific for rabbit IgG conjugated to Alexa Fluor 594 (1:200 Term Fisher Scientific, Waltham, MA, USA, EUA) and with secondary antibodies specific for rabbit IgG conjugated with Alexa Fluor 488 (1:200 Term Fisher Scientific, Waltham, MA, USA, EUA) for 1 h at room temperature. After three washes with PBS, the cells were incubated for 30 min with DAPI for 10 min, and after washing with PBS, the coverslips were mounted on slides with the addition of *N*-propyl gallate to prevent loss of fluorescence. Cell analysis and images were obtained using a fluorescence microscope (LEICA, Wetzlar, Germany, DFC 7000).

### 5.7. Statistical Analysis

Results are expressed as the mean ± standard deviation of the mean (SEM). To determine statistical differences between the groups, an analysis of variance was performed followed by a one-way ANOVA test with Student–Newmann–Keuls post-test (Graph Pad Prisma 5.0—San Diego, CA, USA), to determine the significant differences between the groups that differed in only one parameter. The nonparametric Mann–Whitney test was used to compare two groups. Cell viability was calculated as a percentage with respect to the negative control, which was considered as 100%. “*p*” values less than 0.05 were considered significant.

## Data Availability

The original contributions presented in this study are included in the article. Further inquiries can be directed to the corresponding author.
